# Asymmetrical cross–linguistic semantic activation in Portuguese–English–Chinese trilinguals: evidence from masked translation priming

**DOI:** 10.3389/fpsyg.2025.1734210

**Published:** 2026-01-20

**Authors:** Lishen Yu, Qingqing Kong

**Affiliations:** Faculty of Social Sciences, Humanities and Arts, University of Cape Verde, Praia, Cabo Verde

**Keywords:** L2 mediation, masked translation priming, semantic representation, trilingual processing, typological distance

## Abstract

Research on multilingual semantic representation has largely focused on Indo-European languages, with limited evidence from typologically distant systems. The present study examines Portuguese–English–Chinese trilinguals using a masked translation priming paradigm to investigate whether semantic activation is directionally asymmetrical and how language distance and status influence trilingual processing. Fifty-nine participants completed an animacy judgment task involving 72 non-cognate translation triplets. Reaction times were analyzed with generalized linear mixed-effects models. Results showed significant priming only in the Portuguese → English and English→Chinese directions, while no reliable effects were found in other pairings, including Portuguese ↔ Chinese. These findings suggest that typological distance constrains cross-linguistic activation, whereas L2 can mediate activation toward L3 under conditions of higher proficiency and instructional use. The study highlights typological distance and L2 status as joint determinants of trilingual semantic representation and underscores the need to refine multilingual processing models beyond Indo-European contexts.

## 1 Introduction

Over recent decades, migration-driven demographic change has led many societies toward what Vertovec (2007) terms “super–diversity.” Within this context, interest in multilingualism has grown rapidly, and third language (L3) acquisition has become a focal topic in language education and psycholinguistics (Alidou et al., 2011; Anastassiou et al., 2017; Mouboua et al., 2024). Compared with second language learning, trilingual acquisition poses additional complexities because L3 learners already possess prior linguistic systems that can both facilitate and constrain new learning (Cenoz, 2001; Rothman et al., 2023). As Cenoz and Todeva (2009, p. 278) note, multilinguals often receive “free rides” from previously acquired languages across grammar, pragmatics, vocabulary, pronunciation, and orthography. Becoming multilingual thus entails restructuring of conceptual–linguistic systems rather than the mere accumulation of forms (Kecskes and Zhang, 2009).

A central question for trilingual processing concerns how cross-linguistic semantic activation unfolds during lexical access and whether such activation is directionally asymmetrical (De Bot and Jaensch, 2015). Lexical access engages phonological, orthographic, and semantic representations (Dijkstra, 2009). The lexical–semantic representation refers to the internal system that stores and organizes word meanings via feature-based or network structures, enabling comprehension, retrieval, and cross-linguistic mapping (Collins and Loftus, 1975; Kroll and Stewart, 1994). In trilinguals, activation dynamics may be particularly complex because three lexical–conceptual mappings, degrees of semantic sharing, and control mechanisms interact.

Decades of bilingual research have debated whether two languages share or segregate semantic representations and how mappings between words and concepts are organized (Weinreich, 1953; De Groot and Poot, 1997; Kroll and Stewart, 1994; MacWhinney, 1998; Potter et al., 1984). Converging evidence suggests that while lexical forms can be language-specific, semantic representations are often shared to a considerable extent (De Groot, 1992; Kirsner et al., 2011; Kroll and De Groot, 2014; Salamoura and Williams, 2007; Zeng et al., 2020). Whether such conclusions generalize to trilinguals remains unsettled, and findings frequently reveal directional asymmetries. For instance, in French (L1)–English (L2)–Spanish (L3) trilinguals, masked translation priming appeared robust from L1 to both L2 and L3, but not vice versa (Aparicio and Lavaur, 2016). In Swedish (L1)–English (L2)–Italian (L3), L3 activated L2 but not L1 in word association (Gudmundson, 2020). By contrast, Wang et al. (2023) reported significant priming for L2 → L1, L1 → L3, and L2 → L3, with no reliable effects in the opposite directions in German–English–French trilinguals. Together, these patterns point to partial semantic sharing coupled with direction-specific activation that likely depends on multiple jointly acting factors (e.g., proficiency, acquisition order, and typological distance).

Among these factors, typological distance has received growing attention (Llama et al., 2010; Nelyubina et al., 2025). The Typological Proximity Model proposes that in L3 acquisition, transfer is initially drawn from the previously acquired language most similar to the target (Rothman, 2011, 2015). This perspective predicts that cross–linguistic activation in trilinguals’ mental lexicon is not uniform but biased toward typologically closer sources, with the bias attenuating or shifting as proficiency and usage distributions change.

Mainstream psycholinguistic models have been developed largely on Indo–European languages, leaving East Asian languages under–represented in core theory testing. Given their distinct typological profiles, including tone, morpho–syllabic writing, and different morphosyntactic patterns, studying East Asian targets provides a stringent test of the portability of existing models (Guo and Yuan, 2023). Yet much trilingual work clusters Indo–European combinations (German, French, Norwegian, Italian, Swedish, Polish; Tytus, 2017; Gudmundson, 2020; Lijewska, 2023), where typological proximity complicates the separation of distance effects from proficiency or exposure.

Against this backdrop, the Portuguese–English–Chinese constellation offers a particularly informative testbed. Portuguese and English, while both Indo–European, differ in phonology, inflection, and syntax, and Chinese adds tonal phonology and a morpho-syllabic script, maximizing typological dispersion. This diversity allows us to probe how lexical–semantic activation distributes across distant vs. closer pairings in trilinguals.

The present study investigates asymmetrical cross-linguistic semantic activation in Portuguese–English–Chinese trilinguals using a masked translation priming paradigm, which probes early and relatively automatic access under brief forward-mask conditions (e.g., Forster and Davis, 1984). This paradigm enables us to test whether activation is direction-dependent across three language pairs in both translation directions, and to examine how the observed patterns align with predictions based on typological proximity.

To this end, the investigation addresses two research questions that collectively probe the cognitive architecture of trilingual semantic representation in a typologically diverse system:

RQ1: Do Portuguese–English–Chinese trilinguals exhibit asymmetrical cross-linguistic semantic activation across all six possible translation directions?

RQ2: What role does L2 English play in mediating semantic activation between Portuguese L1 and Chinese L3, particularly in light of the typological distance between these languages and the instructional role of English in Chinese L3 acquisition?

These research questions address critical gaps in trilingual processing research by systematically examining directional asymmetries across typologically diverse languages and testing theoretical predictions concerning L2–mediated cross–linguistic influence. The study aims to provide insights into how Chinese integrates into trilingual systems and to test the generalizability of current theoretical models to language combinations involving typologically distant languages.

## 2 Literature review

### 2.1 Theoretical foundations of cross-linguistic semantic processing

Explanations of asymmetrical cross–language semantic activation in multilingual individuals build on mechanisms originally developed in bilingual research. Two foundational accounts in this literature are the Revised Hierarchical Model (Kroll and Stewart, 1994) and the BIA + framework (Dijkstra and Van Heuven, 2002), both of which offer insights into how asymmetric patterns may arise during lexical access.

The Revised Hierarchical Model proposes that forward translation (L1 → L2) relies primarily on concept mediation, whereas backward translation (L2 → L1) can be accomplished through stronger lexical links. This organization predicts a commonly observed pattern in which L2 → L1 translation is faster and more automatic than L1 → L2, especially among unbalanced bilinguals (Heredia, 1997; Linck et al., 2009; Clenton, 2015; Sperl et al., 2024). The model further suggests that lexical-semantic connection strengths vary as a function of L2 proficiency and L1 dominance, thereby shaping direction–specific processing asymmetries.

Complementing the RHM, the BIA + model assumes nonselective access: words from both languages become co–activated during recognition, with subsequent top–down control mechanisms differentiating between target and non-target languages (Green, 1998; Dijkstra and Van Heuven, 2013; Schwartz and Kroll, 2006). While BIA + successfully explains cross-language co–activation and competition, it remains less explicit about the precise conditions under which directional asymmetries emerge.

Although these bilingual models provide a useful foundation, they do not fully capture the representational and control dynamics inherent in trilingual systems. Trilinguals must coordinate three lexical–semantic networks, navigate more complex competition structures, and manage activation pathways that may involve indirect or cascaded influences across languages. Crucially, one language–often the L2–may possess a special functional status due to differences in learning history, representational format, or instructional role. To address these gaps, researchers have proposed accounts such as the L2 Status Factor Hypothesis (Bardel and Falk, 2007) and the Typological Proximity Model (Rothman, 2011, 2015), both of which aim to characterize how prior linguistic systems differentially influence L3 acquisition and processing.

### 2.2 L2 status factor and typological proximity model: implications for cross-linguistic semantic activation in trilinguals

Bilingual models such as the RHM and BIA + offer valuable insights into cross–language activation but do not fully specify how activation flows across three languages or why particular non–native languages may hold privileged roles in trilingual processing. In L3 acquisition research, two influential frameworks—the L2 Status Factor Hypothesis and the Typological Proximity Model —provide complementary mechanisms for explaining how previously acquired languages shape activation patterns in an emerging L3. Together, these accounts generate testable predictions for semantic activation in typologically diverse systems such as Portuguese–English–Chinese.

#### 2.2.1 L2 status factor hypothesis

The L2 status factor hypothesis (Bardel and Falk, 2007) posits that, in sequential L3 acquisition–where both L2 and L3 are learned in instructional contexts after L1–learners tend to rely more on L2 than on L1 because L2 typically shares similar learning conditions, age of onset, and levels of metalinguistic awareness with L3. This pattern is characteristic of learners who acquire multiple non-native languages through formal education (Bardel and Falk, 2007, 2012; Falk and Bardel, 2011; Angelovska and Hahn, 2012). However, such an idea applies only when L3 is acquired explicitly via mediation in another language.

This privileged role is grounded in three interrelated mechanisms (e.g., Bardel and Falk, 2007; Falk and Bardel, 2011). First, L2 and L3 are typically acquired under similar learning conditions, involving explicit instruction, reliance on metalinguistic knowledge, and intentional learning strategies. This similarity contrasts with the implicit, proceduralized acquisition of L1, making activation between L2 and L3 more likely. Second, L2 and L3 rely more heavily on declarative memory, whereas L1 is largely supported by procedural memory (Bardel and Falk, 2007, 2012). Because non–native languages share representational formats within the declarative system, activation can propagate more efficiently between them during early lexical access, such as under masked priming conditions. Third, the medium-of-instruction effect often strengthens L2–L3 connections. In many L3 Chinese learning environments, English serves as the instructional language for grammar explanations, metalanguage, and classroom communication, leading to frequent pedagogical co-activation of L2 and L3. These experiential factors position L2 as a functional bridge language between the L1 and L3.

Together, these mechanisms predict that in Portuguese–English–Chinese trilinguals, English (L2) should mediate activation toward Chinese (L3), potentially overriding typological distance constraints.

A growing body of trilingual research suggests that an emerging L3 is often accessed through one previously acquired language, and this mediating language is not necessarily the L1 but frequently the L2. Recent masked translation priming evidence indicates that L3 activation is shaped more by learners’ educational history and the language of instruction than by dominance alone. Yang et al. (2025) conducted a large-scale trilingual priming study and demonstrated that L3 English was primed only by L2 Mandarin but not by L1 Korean, even though participants were highly proficient in both languages. Crucially, further analyses showed that this pattern could not be explained by language dominance; instead, the authors argued that formally learned languages tend to become lexically associated with the language through which they were taught. When L3 is acquired in a classroom setting, the instructional language provides the primary representational pathway linking new lexical items to conceptual meaning.

This instruction-mediated account aligns with findings from Aparicio and Lavaur (2016), who reported L3 priming only from the language that served as the medium of instruction, and with masked priming results showing L2 → L3 facilitation even when typological proximity predicts otherwise (Wang et al., 2023). Conversely, L1 → L3 priming is often absent when L1 does not serve as the pedagogical language for L3, even when L1 proficiency is high. The mediating role of L2 is further supported by studies where both L1 and L2 prime L3 only when both previously served as instructional languages or when cognate overlap artificially boosts cross-language activation (Li et al., 2024).

Applied to the present study, these findings are particularly relevant because our participants learned Chinese largely through the medium of English, and they possess relatively strong English proficiency. This learning configuration provides precisely the experiential conditions under which L2 mediation is expected to occur: the L2 becomes the language through which new lexical-semantic mappings in L3 are encoded and retrieved. Thus, prior research predicts—and our experimental results corroborate—that English should facilitate Chinese word recognition, whereas Portuguese, which did not serve as an instructional medium, should not produce such semantic priming effects.

#### 2.2.2 Typological proximity model

The Typological Proximity Model (Rothman, 2011, 2015) attributes cross–linguistic influence to perceived structural similarity between languages. This perspective emphasizes similarities across linguistic domains–lexicon, morphology, syntax, and phonology–as the primary driver of transfer and activation during L3 processing.

Applied to lexical–semantic activation, TPM predicts that typologically closer languages should exhibit: stronger cross–linguistic activation, more symmetric priming, and more efficient mapping between lexical forms and conceptual representations. Within the Portuguese–English–Chinese triad, TPM predicts: strong Portuguese–English activation, reflecting their shared Indo-European origins and overlapping phonological and morphological structures; substantially weaker Portuguese–Chinese activation, given their large typological distance across script, phonology, and morphosyntax. TPM therefore provides a structural explanation for why priming may be asymmetrical or absent between highly distant languages.

#### 2.2.3 Complementary predictions for trilingual semantic activation

Although originally developed to capture different aspects of L3 acquisition, the L2 Status Factor and TPM are best viewed as complementary frameworks. The TPM accounts for influence driven by structural proximity, while the L2 Status Factor explains influence driven by experiential similarity, representational format, and instructional pathways. Together, they offer a dual-mechanism account of semantic activation in trilinguals.

For Portuguese–English–Chinese trilinguals, TPM predicts: Portuguese–English activation, due to structural similarity; weak Portuguese–Chinese and English–Chinese activation, due to strong typological divergence. However, typological distance alone cannot fully explain activation patterns. Both Portuguese–Chinese and English–Chinese are similarly distant typologically, yet empirical evidence consistently shows that English–Chinese activation is more likely than Portuguese–Chinese. This discrepancy suggests that L2-mediated pathways can partially override distance-based constraints. Because English is the L2–acquired through explicit instruction, drawing on declarative memory, and widely used as the medium of L3 Chinese instruction–learners possess experiential and representational conditions that facilitate L2 → L3 activation. Portuguese, lacking these shared properties, does not provide comparable support for activation toward Chinese.

Thus, theoretical predictions for the present language triad are as follows: Portuguese–Chinese priming should be absent or minimal, jointly predicted by TPM and the lack of shared learning conditions proposed by the L2 Status Factor. Portuguese–English priming should be present, consistent with TPM’s structural-similarity account. English–Chinese priming may emerge despite typological distance, reflecting the L2 Status Factor’s proposal that L2 can mediate activation toward a typologically distant L3. These complementary predictions provide a coherent theoretical foundation for interpreting directional asymmetries in the masked semantic priming patterns examined in the present study.

### 2.3 Methodological framework: masked translation priming in trilingual research

Masked translation priming is a widely used paradigm for investigating unconscious cross–linguistic semantic activation while minimizing strategic processing effects. The technique involves presenting prime words for 50–60 ms, followed by pattern masks and target words, enabling researchers to capture automatic activation patterns that vary systematically across language directions (Forster et al., 2003). From a cognitive mechanism perspective, masked priming effects are typically interpreted as indicating that early, unconscious, automatic processing relies primarily on semantic memory systems rather than episodic memory systems, reflecting lexical-level mechanisms rather than low-level sublexical processing driven by visual similarity. This “economical” processing incorporates prime activation into the target’s processing stream upon target presentation, creating advantages for semantically related prime–target pairs (Qiao and Zhang, 2017).

Early masked priming studies used a four-level sequence–a forward mask, a briefly presented prime, a backward mask, and the target (Evett and Humphreys, 1981). Because the prime and the backward mask followed each other within a very short interval, the two sometimes fused perceptually, reducing the effectiveness of the mask. To prevent this fusion effect, later work adopted either a three-level structure (mask → prime → target) or a modified four-level structure that inserts a short blank interval between the prime and the backward mask (Forster and Davis, 1984). The present study uses this modified four-level procedure, which avoids fusion while keeping the prime below conscious awareness, allowing the paradigm to capture early, automatic cross-linguistic activation (Forster et al., 2003; Jiang, 1999; Wen and van Heuven, 2017).

Recent trilingual masked–priming investigations further demonstrate the paradigm’s effectiveness in revealing complex asymmetrical activation patterns. Aparicio and Lavaur (2016) found translation–priming effects only when L1 French served as the prime for L2 English and L3 Spanish targets, with no cross–activation between L2 and L3, suggesting a hierarchical organization privileging the dominant language. A similar dominance–driven pattern was observed by Li et al. (2024), who tested Chinese–English–Japanese trilinguals using triple different-script cognates. Their results showed that priming emerged only when the prime was from the participants’ strongest language (L1 Chinese)—that is, in the L1 → L2 and L1 → L3 directions—while primes from weaker languages (L2 English, L3 Japanese) produced no reliable facilitation. Importantly, Li et al.’s findings indicate that even with phonologically related cognates, cross-language activation requires sufficiently robust prime processing. Together, these studies highlight masked priming’s sensitivity to dominance-based asymmetries that are characteristic of trilingual lexical organization.

Applying masked translation priming to trilingual contexts requires careful attention to methodological factors that distinguish trilingual from bilingual research. First, the six possible language combinations in trilingual systems demand systematic investigation of all translation directions to comprehensively characterize asymmetrical patterns. Second, trilingual proficiency profiles are inherently more complex, necessitating careful control of proficiency differences across the three languages to isolate cross-linguistic activation effects from proficiency-driven differences. Third, potential cascaded activation—for example, Portuguese → English priming secondarily activating Chinese through English’s role as instructional medium—necessitates designs that can separate direct cross-linguistic effects from indirect L2-mediated pathways. Fourth, multilingual contexts may introduce additional sources of interference that warrant consideration; for instance, Toassia and Motab (2018) demonstrated L2 German interference in L3 English production among Portuguese natives, indicating that non-target languages can influence trilingual processing even when not directly manipulated.

### 2.4 Empirical evidence for trilingual semantic activation

The precise nature of these asymmetries varies substantially across studies, resulting in a complex empirical landscape that must be carefully examined to address our research questions on Portuguese-English-Chinese trilingual processing.

Evidence from hierarchical organization studies demonstrates clear L1-privileged patterns. Aparicio and Lavaur (2016) examined French-English-Spanish trilinguals using masked translation priming with 67 ms prime duration, reporting significant priming effects only when L1 French served as the prime for both L2 English and L3 Spanish targets, with no L2–L3 interaction. This suggests that L1 retains privileged access to both non-native languages, while L2 and L3 remain functionally isolated—directly relevant to RQ1 concerning asymmetrical activation patterns. Abunuwara’s (1992) Stroop experiments with Arabic-Hebrew-English trilinguals corroborated this hierarchical view, showing interference effects between L1 and both L2 and L3, but no L2–L3 interference. More recently, Ashraf (2025) confirmed this pattern in Punjabi-Urdu-English trilinguals across picture-naming, Stroop, and translation tasks, demonstrating stronger L1 activation effects compared to L2 in multiple experimental contexts.

In contrast, research supporting partial semantic integration reveals more complex activation patterns that challenge simple hierarchical models. Wang et al. (2023) conducted a systematic investigation of German-English-French trilinguals, providing comprehensive evidence for directional asymmetries directly relevant to our research questions. Using masked translation priming with a 50 ms prime duration across 8 participants, they found significant priming effects in the L2–L1, L1–L3, and L2–L3 directions, but no effects for L1–L2, L3–L1, or L3–L2. Crucially, their modified Sense Model framework showed that language dominance, rather than acquisition order, determined priming strength, with evidence of dynamic dominance shifts between L1 and L2 during lexical development.

Cross-linguistic influence research also offers essential insights into the mechanisms underlying trilingual vocabulary processing. Mulík et al. (2019) studied Spanish-English bilinguals learning L3 Slovak using paired-associate tasks and found comparable facilitation effects for both L3–L1 and L3–L2 phonological similarity, even when L2 was not explicitly present in the task. However, participants with higher L2 proficiency showed greater benefits from L2 similarity, indicating that L2 activation strength correlates with proficiency. Extending this work, Mulík and Carrasco-Ortiz (2023) used ERP methods and revealed divergent neurocognitive mechanisms for L3 learning via L1 versus L2, showing opposite N400 effects for L1 Spanish and L2 English interlingual homophones after only 3 days of training. This points to distinct pathways of cross-linguistic influence. Otwinowska (2024) synthesized mounting evidence for cumulative cross-linguistic influence in L3 vocabulary acquisition across multiple paradigms. Analyzing L3 word processing experiments, cognate guessing tasks, and vocabulary learning studies, she demonstrated that triple cognates (shared across L1, L2, and L3) consistently provide processing advantages over double cognates, provided that L2 and L3 proficiency reaches threshold levels.

Taken together, these empirical findings establish three key patterns relevant to the present study. First, directional asymmetries are a consistent feature of trilingual processing, though their manifestations vary systematically across language combinations and proficiency profiles. Second, L2 demonstrates unique activation characteristics that distinguish it from both L1 and L3, supporting theoretical accounts of L2’s mediating role in trilingual systems. Third, typological distance significantly shapes cross-linguistic activation strength, with typologically closer pairs showing different facilitation patterns than more distant pairs.

Nevertheless, current research suffers from a critical limitation: the overwhelming focus on Indo-European language combinations restricts our understanding of how typological diversity modulates trilingual processing mechanisms. The present study addresses this gap by examining Portuguese-English-Chinese trilinguals, thereby enabling a systematic investigation of how typological distance influences asymmetrical activation patterns across all translation directions while testing theoretical predictions concerning L2-mediated cross-linguistic influence.

## 3 Methods

### 3.1 Participants

Fifty-nine Portuguese–English–Chinese trilinguals (36 female, 23 male; *M* = 30.17 years, SD = 6.95, range = 20–45 years) participated in the study. Inclusion criteria required native Portuguese speakers who had received formal instruction in both English and Chinese as foreign languages. All were right-handed, had normal or corrected-to-normal vision, and none reported any history of neurological disorders.

Language acquisition patterns showed that English (L2) was acquired significantly earlier (*M* = 11.31 years, SD = 2.67) than Chinese (L3; *M* = 19.95 years, SD = 6.05), *t*(58) = −10.55, *p* < 0.001, *d* = 1.37. Both foreign languages were learned primarily through classroom instruction rather than naturalistic immersion. Self-rated proficiency, measured on a 7-point Likert scale (Li et al., 2020), indicated significantly higher competence in English (*M* = 5.71, SD = 0.96) than in Chinese (*M* = 4.18, SD = 1.27) across all language modalities, *t*(58) = 7.85, *p* < 0.001, *d* = 1.38.

Table 1 presents detailed proficiency ratings across the four skill domains. Effect size analyses showed large differences between English and Chinese in all skills (Cohen’s *d* > 1.40), with English consistently rated higher. Portuguese remained the dominant language for daily communication and cognitive processing, English was primarily used in professional and academic contexts, and Chinese was mainly restricted to classroom learning and limited social interactions with Chinese speakers.

**Table 1:** Language proficiency ratings by skill domain.

Language skill	English		Chinese		*t*(58)	*p*	*d*
	*M*	SD	*M*	SD			
Listening	5.86	0.89	4.59	0.89	7.81	<0.001	1.43
Speaking	5.71	1.03	4.25	0.79	8.42	<0.001	1.58
Reading	5.85	0.90	4.24	1.05	8.92	<0.001	1.66
Writing	5.42	1.12	3.63	0.90	9.42	<0.001	1.75
Overall	5.71	0.96	4.18	1.27	7.85	<0.001	1.38

### 3.2 Materials

Seventy two Portuguese–English–Chinese translation triplets were selected to ensure cross-linguistic comparability. All items were concrete nouns that met three criteria: (1) high familiarity ratings across all three languages (≥ 6.0 on a 7-point scale), (2) absence of cognate relationships between Portuguese and English to avoid orthographic confounds, and (3) availability of clear translation equivalents in Chinese. Half of the stimuli denoted animate entities (animals, professions), and half represented inanimate objects (tools, furniture), conforming to binary animacy judgment requirements.

Portuguese and English words were matched for length, with Portuguese items averaging 5.97 letters (SD = 1.84) and English items averaging 5.74 letters (SD = 1.92), *t*(143) = 0.84, *p* = 0.401. Chinese stimuli were two-character compounds presented in simplified script to ensure semantic transparency and avoid the processing complexity associated with single-character presentations.

Prior to the experiment, two Portuguese-English-Chinese trilinguals who did not participate in the main study independently rated the familiarity of candidate words on a 7-point scale (1 = very unfamiliar, 7 = very familiar). Words were included in the final stimulus set only if both raters gave a score of 6 or above. The mean familiarity ratings of the final word set were 6.45 (SD = 0.38) for Portuguese, 6.52 (SD = 0.35) for English, and 6.48 (SD = 0.41) for Chinese, ensuring high familiarity and translation accuracy across all three languages.

#### 3.2.1 Word-frequency control

Because word frequency is a robust predictor of lexical processing (Brysbaert et al., 2018), primes and targets were matched on frequency using subtitle-based corpora: SUBTLEX-US for English (Brysbaert and New, 2009), SUBTLEX-PT for Portuguese (Soares et al., 2015), and SUBTLEX-CH for Chinese (Cai and Brysbaert, 2010). Subtitle-based norms typically outperform older written-corpus measures in predicting lexical decision and naming latencies because they more closely approximate everyday language exposure (Brysbaert and New, 2009). Frequencies are reported as occurrences per million words.

Across conditions, descriptive statistics were as follows. In the related condition, primes had a mean frequency of 66.35 per million (SD = 97.43, range = 1.85–609.74), and targets had a mean frequency of 77.82 per million (SD = 112.74, range = 2.20–529.82). In the unrelated condition, primes had a mean frequency of 49.58 per million (SD = 66.96, range = 0.22–330.02), and targets had a mean frequency of 99.20 per million (SD = 180.60, range = 0.14–933.34).

To assess frequency matching formally, we conducted paired-samples *t*-tests comparing prime and target frequencies within each condition, with frequencies averaged across item sets within each translation direction. This aggregation explains the reported degrees of freedom in the paired comparisons. No significant differences were observed in the related condition, *t*(23) = 1.21, *p* = 0.240, nor in the unrelated condition, *t*(20) = −1.64, *p* = 0.117. Although mean frequencies in the unrelated condition appear numerically different, frequency distributions were highly skewed and characterized by substantial variance, resulting in non-significant paired comparisons.

Independent-samples t-tests comparing related and unrelated stimuli also yielded non-significant results for primes, *t*(91) = 0.97, *p* = 0.337, and for targets, *t*(93) = −0.69, *p* = 0.489, indicating that frequency distributions did not differ systematically across conditions. Taken together, these checks indicate that the observed priming effects cannot be readily attributed to frequency confounds.

#### 3.2.2 Construction of unrelated pairs

For the unrelated condition, we constructed a new set of prime–target pairings by re-pairing lexical items drawn from the same stimulus pool used in the related condition, rather than introducing entirely new lexical items. This procedure ensured that related and unrelated conditions were comparable at the item level, while eliminating semantic and translation relationships between primes and targets.

Unrelated items were selected and paired under the following constraints: (a) Word frequency: prime and target words in the unrelated condition did not differ significantly in frequency from those in the related condition; (b) Animacy: unrelated pairs respected the same animacy constraints as the related pairs (animate–animate or inanimate–inanimate); (c) Lexical features: unrelated primes and targets were matched to related items in length, part of speech (all concrete nouns), and—where applicable—syllable or character count; (d) Semantic relationship: primes and targets in unrelated pairs were entirely unrelated in meaning and shared no translation equivalence. This procedure ensured that related and unrelated conditions were closely matched on key lexical variables (e.g., frequency, animacy, word class), while differing only in the presence versus absence of semantic and translation relationships between primes and targets.

For each of the six translation directions, 24 experimental trials were constructed: 12 translation-related pairs (6 animate, 6 inanimate) and 12 unrelated control pairs, created by re-pairing items while maintaining animacy balance. This design yielded 144 critical trials per participant (6 directions × 24pairs), providing sufficient statistical power to detect cross-linguistic priming effects without unduly prolonging the experimental session.

### 3.3 Design and procedure

A within-subjects design was used to examine masked translation priming across six translation directions: Portuguese↔English (PT → EN, EN → PT), Portuguese↔Chinese (PT → ZH, ZH → PT), and English↔Chinese (EN → ZH, ZH → EN). This comprehensive design allowed systematic examination of bidirectional priming across all language pairs, essential for detecting asymmetrical cross-linguistic semantic activation.

Testing took place individually in acoustically isolated rooms, with E-Prime 3.0 used for precise timing control. Each trial followed a standardized masked priming sequence: forward mask (######, 500 ms) → prime (100 ms) → blank interval (50 ms) → backward mask ($$$$$$, 50 ms) → target (displayed until response or maximum 2,500 ms). The 200 ms stimulus onset asynchrony (SOA) was chosen based on prior findings that this interval captures automatic semantic processing while minimizing conscious translation strategies (Forster and Davis, 1984).

**Figure 1:**
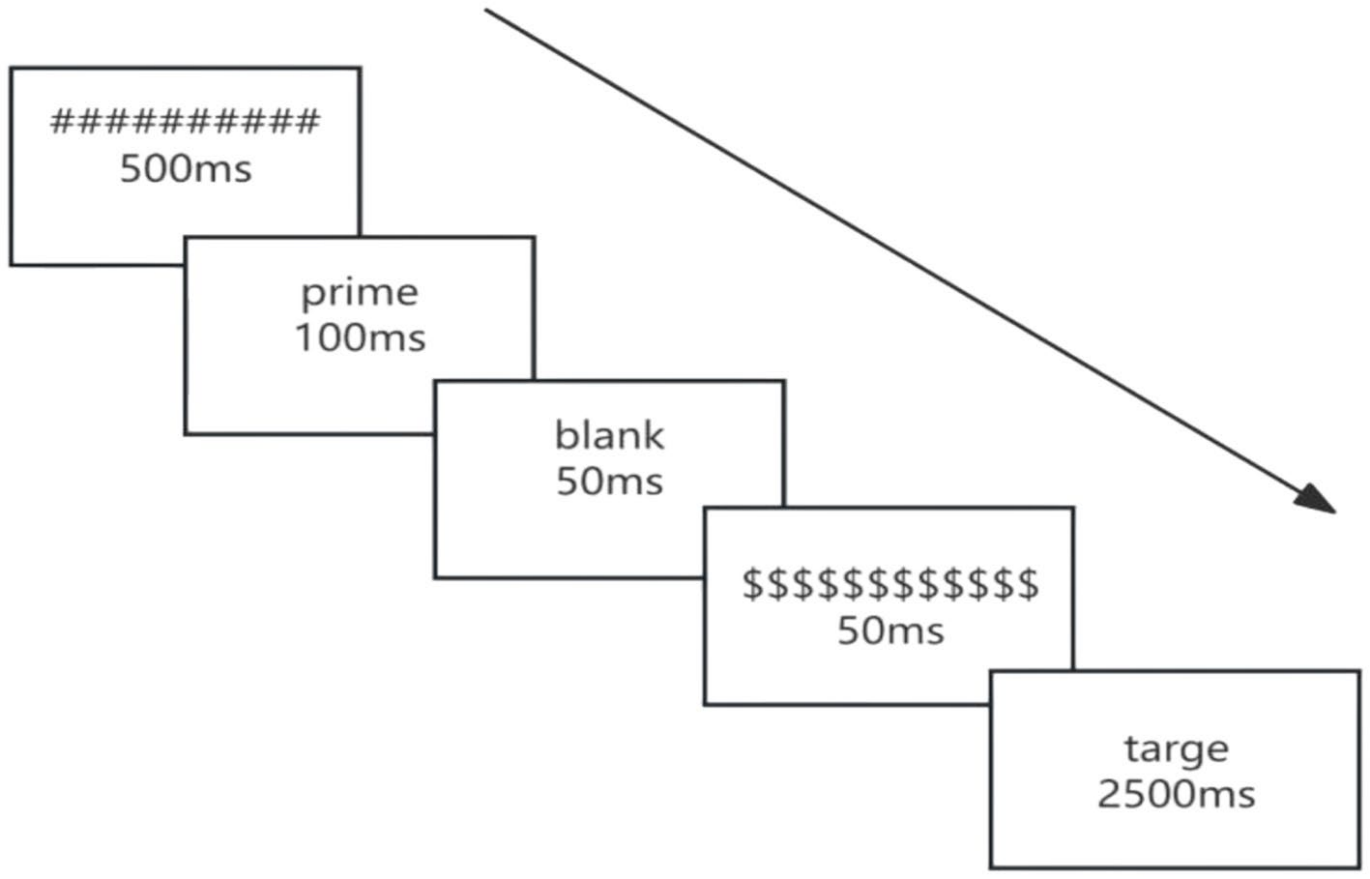
Schematic representation of a single trial sequence in the masked translation priming paradigm.

Stimuli appeared in uppercase against a white background. Portuguese and English words were displayed in 24-pt Arial, and Chinese characters in 28-pt SimHei, to ensure comparable visual salience across scripts. Participants performed animacy judgments using designated response keys (“A” = animate, “L” = inanimate), with key-mapping counterbalanced across participants to control for motor biases. The animacy judgment task was selected to ensure semantic processing of targets without explicitly encouraging translation strategies, providing an appropriate measure of unconscious cross-linguistic activation. Reaction times were recorded from the onset of the target display until the participant’s keypress response (Figure 1).

After six practice trials with feedback, participants completed 144 randomized experimental trials presented without feedback to avoid learning effects. No breaks were given to maintain consistent testing conditions. At the end of the session, participants completed a comprehensive language history questionnaire (Li et al., 2020). Each session lasted approximately 30 min. All participants gave written informed consent.

### 3.4 Power analysis

To justify the adequacy of the experimental design, we followed established recommendations in masked priming research indicating that statistical power in within-subject RT paradigms is primarily determined by the number of trials rather than the number of participants (Brysbaert and Stevens, 2018). Each participant completed 144 critical trials (24 per translation direction), yielding a dense trial structure that increases sensitivity to small priming effects in generalized linear mixed-effects models.

According to best-practice guidelines for reaction-time experiments (Brysbaert and Stevens, 2018), designs with ≥ 1,600 observations per fixed-effect comparison typically achieve ≥ 80% power to detect small effects (*d* ≈ 0.15–0.20). In the present study, each direction-specific GLMM included between 1,416 and 1,472 usable RT observations after trimming, closely approaching the recommended threshold. Together, the within-subject manipulation, the high number of item-level observations, and the use of mixed-effects modeling provide adequate statistical power to detect masked priming effects of the magnitude commonly reported in bilingual and trilingual studies.

### 3.5 Statistical modeling framework

Reaction times were analyzed using generalized linear mixed-effects models (GLMMs) implemented in the lme4 package in R (Bates et al., 2015). Because RT distributions are positively skewed, models were fitted with a Gamma distribution and a log link, which provides a better distributional fit than Gaussian models in masked priming paradigms. For each language pair, the following model was specified: RT ∼ PrimingType∗Direction+(1∣Participant) + (1∣Item).

PrimingType (related vs. unrelated) was contrast-coded (−0.5 = related, +0.5 = unrelated).

#### 3.5.1 Definition of the direction variable

Direction refers to the directionality of translation between the two languages tested in each experiment. It was coded as a binary factor with two levels: forward: L1 → L2 or L2 → L3backward: L2 → L1 or L3 → L2. This variable captures potential asymmetries arising from whether the prime comes from a more dominant language (L1), the second language (L2), or the least dominant language (L3). Thus, Direction models whether priming differs depending on which language serves as the prime versus the target within the same language pair, independent of the priming manipulation itself. Direction was contrast-coded (−0.5 = forward, +0.5 = backward).

#### 3.5.2 Random-effects structure

Random intercepts were included for Participants and Items. Random slopes were not included: with only 24 items per translation direction, maximal random-effects structures produced singular fits and unstable variance estimates. Following recommendations by Matuschek et al. (2017), a parsimonious random-intercept structure was adopted to ensure model convergence, stability, and appropriate Type I error control in masked priming designs with tightly controlled stimuli. Significance of fixed effects was assessed using likelihood ratio tests comparing models with and without the effect of interest.

#### 3.5.3 Planned simple-effects analyses

Following established practice in masked translation priming research, where directional asymmetries are a central theoretical phenomenon (Wang and Forster, 2010; Aparicio and Lavaur, 2016), we conducted planned simple-effects analyses for each translation direction within all language pairs. Prior studies have consistently demonstrated that robust directional effects can emerge even when omnibus interactions fail to reach significance (e.g., Wang and Forster, 2010, Experiment 1), necessitating direction-specific planned comparisons (Aparicio and Lavaur, 2016) to fully characterize cross-linguistic activation patterns and test theoretical predictions regarding asymmetric semantic connectivity.

## 4 Results

### 4.1 Unified mixed-effects model

We first fitted a unified mixed-effects model to reaction times, including Language Pair, Direction, and Relatedness as fixed effects, as well as all corresponding interactions. Random intercepts were specified for participants and items, with by-participant random slopes for Relatedness. The model was fitted using a Gamma distribution with a log link function and converged successfully without singular fits (*N* = 610).

The model revealed significant baseline differences in reaction times across language pairs, as well as a significant overall asymmetry between forward and backward translation directions. The main effect of Relatedness did not reach significance, and the LanguagePair × Relatedness interaction was also not significant. Fixed effects from the unified model are summarized in Table 2.

**Table 2:** Mean reaction times and cross-linguistic priming effects by translation direction.

Direction	Language pair	Related RT	Unrelated RT	Priming effect
L1 → L2	Portuguese → English	729.32 (142.30)	743.17 (156.80)	+13.85*
L2 → L1	English → Portuguese	735.60 (138.70)	730.98 (144.20)	−4.62
L1 → L3	Portuguese → Chinese	805.58 (167.40)	815.69 (172.90)	+10.11
L3 → L1	Chinese → Portuguese	694.05 (129.60)	726.49 (147.30)	+32.44
L2 → L3	English → Chinese	809.87 (164.20)	927.59 (189.70)	+117.72***
L3 → L2	Chinese → English	758.02 (151.80)	775.21 (158.40)	+17.19

Values in parentheses are standard deviations. Priming effect = Unrelated RT – Related RT; positive values indicate facilitation. *p < 0.05, ***p < 0.001.

Given the study’s theoretical focus on directional asymmetries in trilingual lexical access, we next conducted planned follow-up analyses examining simple effects of Relatedness within each translation direction. Fixed effects from the unified model are summarized in Table 2.

### 4.2 Data preparation and descriptive statistics

Data were preprocessed following standard procedures for masked priming studies. The goal of this section is to provide an overview of the descriptive patterns that motivate the subsequent mixed-effects analyses. We report mean reaction times (RTs) and priming effects for each translation direction to illustrate the overall asymmetry pattern before turning to the inferential statistics. These descriptive results allow readers to understand the direction and magnitude of the effects observed in the GLMMs.

No participants were excluded based on overall accuracy, as all participants responded correctly on more than 80% of trials. Trials with incorrect animacy judgments were removed (6.8% of trials). Reaction times below 200 ms or exceeding±2.5 standard deviations from each participant’s mean were further excluded (2.1% of remaining trials). In total, 91.1% of the original dataset was retained for analysis. Table 2 presents mean RTs and priming effects (unrelated RT–related RT, where positive values indicate facilitation) for each translation direction. Overall latencies varied systematically across target languages, with Chinese as target producing longer RTs (Portuguese → Chinese: 811 ms; English → Chinese: 870 ms) compared to Portuguese or English targets (694–758 ms). Priming effects revealed marked directional asymmetries, ranging from slight inhibition in English → Portuguese (−4.62 ms) to substantial facilitation in English → Chinese (+117.72 ms).

### 4.3 Statistical analysis

Data were analyzed using generalized linear mixed-effects models (GLMMs) with a Gamma distribution and log link function to accommodate positively skewed RT distributions. Models were implemented with the lme4 and lmerTest packages in R (Bates et al., 2015; Kuznetsova et al., 2017). Following conventions in trilingual priming research, separate models were fit for each language pair to facilitate clearer interpretation of directional effects. This approach avoids conflating cross-pair variability arising from differences in script, baseline RT, or distributional properties across languages. It also allows each pair to be modeled with an appropriate variance structure, improving interpretability while maintaining comparability across models.

Each model included fixed effects of Priming Type (related vs. unrelated, contrast-coded), Direction (forward vs. backward, contrast-coded), and their interaction. Random intercepts were specified for participants and items. Model parameters were estimated using maximum likelihood, and significance was assessed with likelihood ratio tests (Table 3).

**Table 3:** Fixed effects from the unified mixed-effects model.

Fixed effect	*β*	*z*	*p*
Language pair (L1–L2 vs. others)	−0.055	−3.35	<0.001
Language pair (L1–L3 vs. L2–L3)	−0.042	−2.12	0.034
Direction (Forward vs. Backward)	0.038	2.42	0.016
Relatedness (Related vs. Unrelated)	−0.024	−1.54	0.124
LanguagePair × Relatedness	—	—	>0.89

Reaction times were analyzed using a generalized linear mixed-effects model with a Gamma distribution and a log link function. Fixed effects included Language Pair, Direction, and Relatedness. Random intercepts were specified for participants and items, with by-participant random slopes for Relatedness. Regression coefficients (β) are reported on the log scale. For Language Pair, planned contrasts compared (i) L1–L2 against the average of the other language pairs (L1–L3 and L2–L3), and (ii) L1–L3 against L2–L3.

### 4.4 Language-pair specific analyses

We first examined the Portuguese–English pair to establish whether masked semantic priming occurs between the participants’ dominant language (L1) and their highly proficient L2. Results showed a significant main effect of Priming Type, *χ*^2^(1) = 4.14, *p* = 0.042, indicating that targets preceded by translation-related primes were judged faster than those preceded by unrelated primes, consistent with automatic semantic facilitation in masked priming. Neither Direction, *χ^2^*(1) = 0.22, *p* = 0.638, nor the interaction, *χ^2^*(1) = 2.50, *p* = 0.114, reached significance. Simple effects analysis indicated significant facilitation for Portuguese→English (+13.85 ms), *z* = −2.04, *p* = 0.042, Cohen’s *d* = 0.06, although the effect size was negligible. By contrast, English → Portuguese showed no reliable effect (−4.62 ms), *z* = −0.01, *p* = 0.991, *d* = −0.02.

We next turned to the Portuguese–Chinese pair to assess whether priming emerges between the typologically most distant languages in the trilingual system. The analysis revealed a significant main effect of Direction, *χ^2^*(1) = 6.12, *p* = 0.013, reflecting overall RT differences between directions. Neither Priming Type, *χ^2^*(1) = 0.25, *p* = 0.617, nor the interaction, *χ^2^*(1) = 0.15, *p* = 0.696, reached significance. Simple effects showed no reliable priming in either direction: Portuguese → Chinese (+10.11 ms), *p* = 0.617, *d* = 0.03, or Chinese → Portuguese (+32.44 ms), *p* = 0.292, d = 0.16. Although the numerical difference was slightly larger in the Chinese → Portuguese direction, the effect did not reach significance.

Finally, we analyzed the English–Chinese pair to determine whether L2 facilitates access to L3, as predicted by accounts proposing a mediating role of the non-native language. This language pair produced the strongest semantic facilitation effects, with markedly faster responses for targets preceded by translation-related primes. There was a robust main effect of Priming Type, *χ^2^*(1) = 11.86, *p* < 0.001, and a marginal Priming Type × Direction interaction, *χ^2^*(1) = 3.77, *p* = 0.052. Simple effects revealed robust facilitation in English → Chinese (+117.72 ms), *z* = −3.44, *p* < 0.001, Cohen’s *d* = 0.30, representing a medium effect size. By contrast, Chinese → English showed no reliable priming (+17.19 ms), *z* = −0.70, *p* = 0.484, *d* = 0.09 (Table 4).

**Table 4:** Statistical results of cross-linguistic priming analyses.

Direction	Language pair	Effect (ms)	*z-ratio*	*p*	Cohen’s *d*	95% CI
L1 → L2	Portuguese → English	+13.85	−2.04	0.042*	0.06	[−0.07, 0.19]
L2 → L1	English → Portuguese	−4.62	−0.01	0.991	−0.02	[−0.14, 0.10]
L1 → L3	Portuguese → Chinese	+10.11	−0.50	0.617	0.03	[−0.09, 0.15]
L3 → L1	Chinese → Portuguese	+32.44	−1.05	0.292	0.16	[0.05, 0.27]
L2 → L3	English → Chinese	+117.72	−3.44	<0.001***	0.30	[0.17, 0.42]
L3 → L2	Chinese → English	+17.19	−0.70	0.484	0.09	[−0.06, 0.24]

Results from GLMM analyses with Gamma distribution. *p < 0.05; ***p < 0.001.

### 4.5 Summary of cross-linguistic semantic priming effects

This section synthesizes the results across all six translation directions and highlights the overarching pattern of cross-linguistic semantic priming observed in the dataset. Across all six translation directions, only two showed significant priming: English → Chinese (*p* < 0.001) and Portuguese → English (*p* = 0.042). All other directions failed to reach significance. Here, “priming” refers specifically to faster animacy judgments for targets following translation-related primes, reflecting early automatic semantic activation rather than explicit translation or language-level activation.

**Figure 2:**
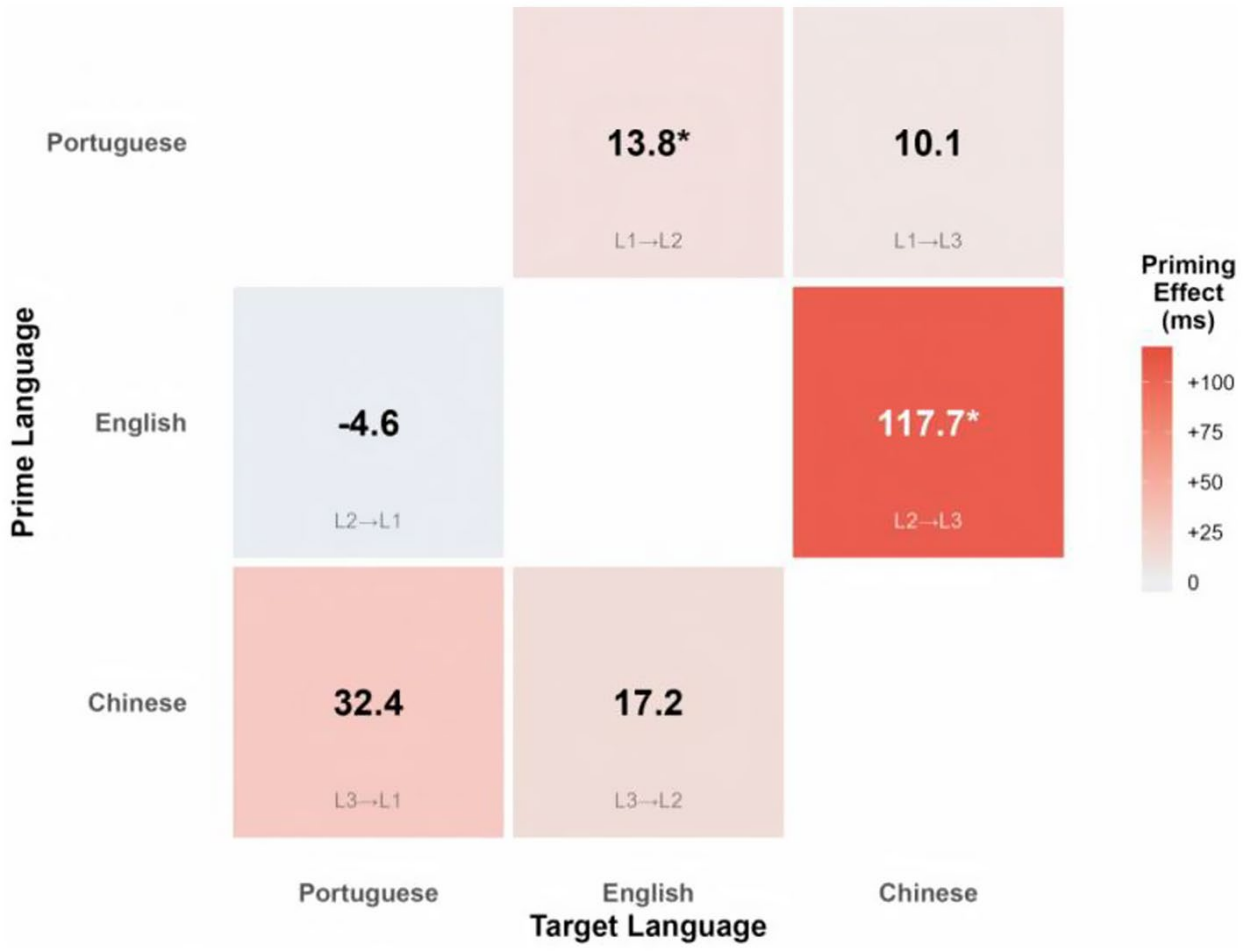
Cross-linguistic semantic priming effect matrix. Priming effects (ms) were calculated as unrelated RT –related RT. Positive values (red shading) indicate facilitation; significant effects are marked with asterisks (*).

**Figure 3:**
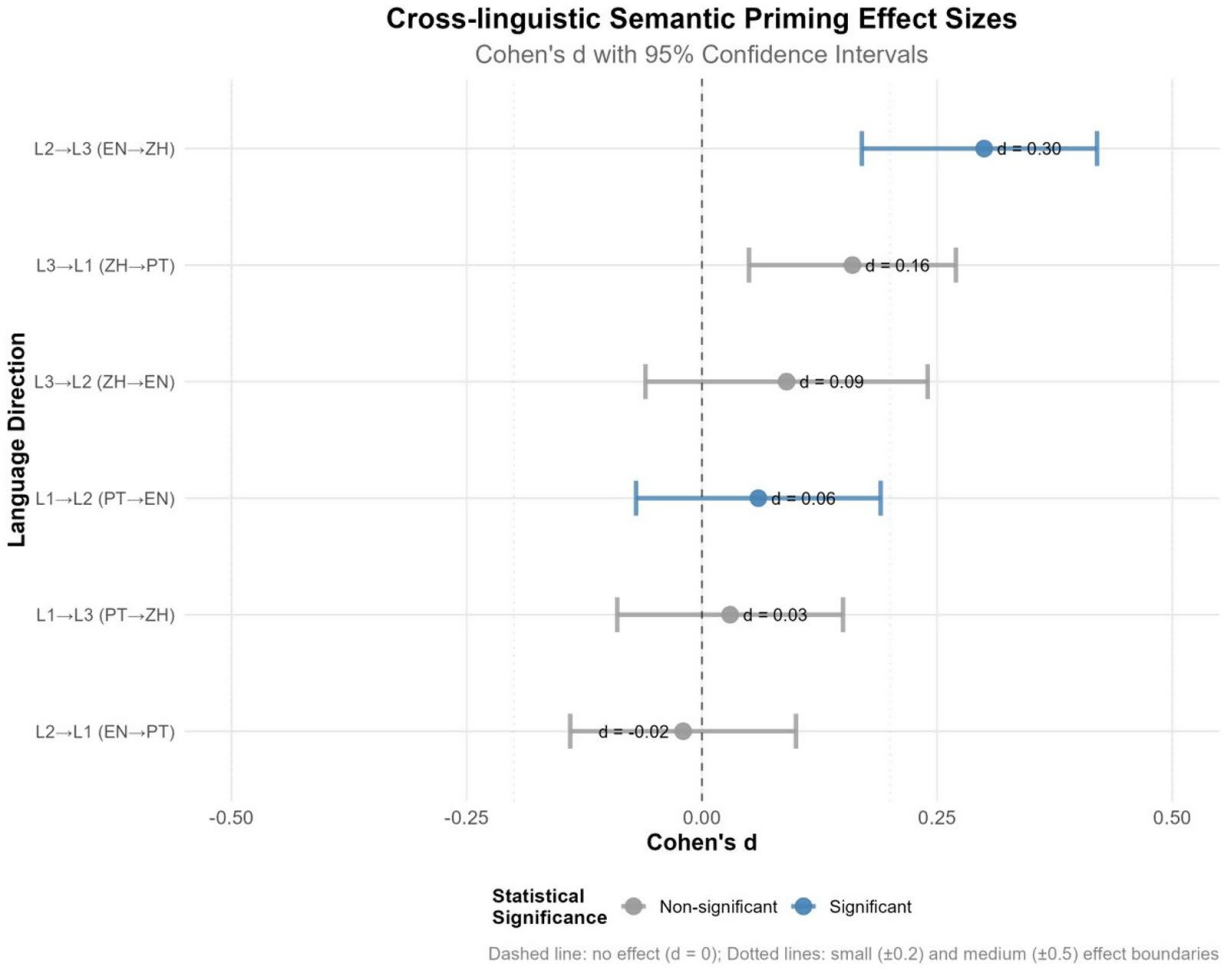
Cross-linguistic semantic priming effect sizes. Forest plot of Cohen’s *d* with 95% confidence intervals. Blue markers indicate significant effects (*p* < 0.05); gray markers indicate non-significant effects. The dashed line at *d* = 0 denotes no effect; dotted lines represent conventional small (±0.2) and medium (±0.5) boundaries.

As shown in Figure 2, the heatmap of priming effects (ms) illustrates the asymmetrical pattern, with robust facilitation in the L2 → L3 (English → Chinese) direction and weaker or null effects elsewhere. Figure 3 presents a forest plot of effect sizes (Cohen’s *d* with 95% confidence intervals). Both figures converge in demonstrating reliable priming for English → Chinese and Portuguese → English, but not in other directions.

## 5 Discussion

### 5.1 Directional asymmetries in cross-linguistic semantic activation

The present study revealed a clear asymmetrical pattern of cross-linguistic semantic activation among trilingual speakers, with significant priming effects observed exclusively in the English → Chinese and Portuguese → English directions, while other language combinations failed to produce reliable effects. This directional asymmetry directly addresses RQ1 and provides important insights into the organizational principles of trilingual semantic representation.

The Portuguese → English effect aligns with the predictions of the Revised Hierarchical Model (RHM), which posits that L1 lexical items maintain direct conceptual connections, whereas L2 access is mediated through L1 (Kroll and Stewart, 1994). This explains why Portuguese primes facilitated English targets, while the reverse direction did not. Extensive bilingual research corroborates this asymmetry, consistently demonstrating robust L1 → L2 priming but weak or absent L2 → L1 effects across both similar and distinct writing systems (De Groot and Nas, 1991; Voga and Grainger, 2007; Tomaz et al., 2024).

The Bilingual Interactive Activation Plus (BIA+) model (Dijkstra and Van Heuven, 2002) provides a complementary account by emphasizing differential resting activation levels. Portuguese as L1 maintains higher baseline activation, enabling rapid conceptual activation and spreading to L2. By contrast, English as L2 operates at a lower resting activation level, limiting its ability to facilitate L1 targets under masked conditions.

The significant English → Chinese effect is particularly noteworthy, as it challenges traditional assumptions of L1 dominance. It aligns with evidence showing that L2 can function as a gateway to L3 when proficiency is high or when L2 assumes functional dominance (Chen, 2020). Pathak et al. (2025) similarly reported that dominant L2s facilitated faster L3 processing in multilinguals. These observations are consistent with the Shared Distributed Asymmetry Model, which proposes that L2 exerts stronger influence on L3 than vice versa, reflecting acquisition sequence rather than strict L1 dominance.

The present findings demonstrate both convergence and divergence with prior trilingual research. The Portuguese → English asymmetry shows strong continuity with bilingual traditions, replicating forward priming effects documented across multiple language pairs (Bailey et al., 2024; Dimitropoulou et al., 2011). However, notable differences emerge in trilingual contexts.

A closely related line of evidence comes from Li et al. (2024), who examined masked translation priming in Chinese–English–Japanese trilinguals using triple different-script cognates. Their results revealed a strong dominance-driven asymmetry: significant priming emerged only when primes originated from the participants’ strongest language (L1 Chinese), whereas L2 and L3 primes produced no reliable effects. Importantly, even with phonologically related cognates—which typically yield larger priming effects—the weaker languages (English and Japanese) failed to prime the stronger one, underscoring the necessity of robust prime activation for cross-language facilitation. This pattern contrasts with the present study in two respects. First, our use of non-cognate translation pairs isolates semantic activation from phonological similarity, allowing us to show that the same dominance-driven asymmetry arises even without cognate facilitation. Second, unlike Li et al.’s demonstration of strong L1 → L2/L3 effects, our Portuguese → Chinese and Chinese → Portuguese pairs did not produce priming, highlighting the additional constraining role of typological distance rather than dominance alone.

Aparicio and Lavaur (2016) examined unbalanced French (L1)–English (L2)–Spanish (L3) trilinguals and found that significant priming occurred only when primes originated from the dominant language (L1). Specifically, L1 primes significantly accelerated recognition of both L2 and L3 targets, suggesting that multilingual mental lexicons may be organized around L1, with limited direct interaction between non-dominant languages (L2 and L3). By contrast, the absence of Portuguese → Chinese and Chinese → Portuguese priming in the present study diverges from reports of observable L1–L3 activation in some trilingual populations.

Wang et al. (2023) identified significant L1–L3 priming in German–English–French trilinguals, whereas Tytus (2017), using semantic categorization tasks, reported bidirectional L1–L2 priming but no L1–L3 effects. Such cross-study patterns suggest that L3-to-L1 priming is generally weak, particularly when proficiency is low or typological distance is large.

Nevertheless, some studies have reported different representational patterns. Chen and Zhang (2012), for example, found that Cantonese–Mandarin–English trilinguals shared a common semantic representation across all three languages, with strong conceptual connections between each pair. This stands in sharp contrast to the directional asymmetries observed here. However, several features of their sample warrant caution: Cantonese is a linguistic variant of Mandarin with high similarity; participants were raised in Mandarin-speaking environments with formal education; and all had passed the College English Test Band 6. These factors render them closer to highly proficient bilinguals than prototypical trilinguals, which may explain their symmetric representation pattern.

Similarly, Dilraba (2012) investigated Uyghur trilinguals using cross-linguistic long-term repetition priming in category judgment tasks and found that when L3 proficiency was high, L3 could effectively prime L2. By contrast, in the present study, L3 (Chinese) was the last-acquired, least-proficient, and least-used language, which likely contributed to the absence of significant L3 → L2 priming effects. Task paradigms may also account for discrepancies: Wang (2016) showed that semantic categorization tasks are more likely to elicit cross-linguistic priming, whereas lexical decision tasks often produce weaker or null L3 → L2 effects.

Taken together, these comparative findings highlight that typological distance, proficiency, and task paradigm jointly shape cross-linguistic activation in trilinguals. They also suggest that Portuguese–English–Chinese trilinguals represent a unique configuration that both aligns with and challenges models developed primarily from Indo-European language combinations.

### 5.2 Typological distance effects and L2 mediation

Typological distance and language status are widely recognized as key determinants of cross-linguistic influence in third language acquisition, with effects documented across lexical, morphosyntactic, semantic, and phonological domains (Hammarberg, 2001; Chiswick and Miller, 2005). Typological distance encompasses both objective structural differences and psychotypology, learners’ subjective perceptions of similarity (Jaekel et al., 2024). Research shows that learners often rely primarily on lexical similarity when judging relatedness, rather than phonological or syntactic overlap (Rothman, 2015). When subjective perceptions align with objective typology, cross-linguistic transfer and integration are more likely to occur (Ringbom, 2007; Singleton and Ó Laoire, 2006).

Portuguese and Chinese, as cross-family languages, are typologically distant. Portuguese, a Romance language within the Indo-European family, is characterized by rich inflectional morphology, whereas Chinese, a Sino-Tibetan language, relies on word order and particles rather than inflectional morphology. Their orthographic systems further accentuate this divide: Portuguese employs an alphabetic script, whereas Chinese uses morphosyllabic characters. Jiang’s (2000) psycholinguistic model suggests that typological distance constrains lexical integration. When languages are typologically close, L2 vocabulary is more likely to achieve full semantic integration; when distant, lexical representations often remain partially integrated, maintaining relative separation. This framework accounts for the absence of Portuguese ↔ Chinese priming in the present study. From a psychotypological perspective, Portuguese speakers may perceive Chinese as highly incompatible with Romance languages, thereby raising activation thresholds and reducing the likelihood of cross-language priming under masked conditions.

By contrast, Portuguese and English, as Indo-European languages, share abundant cognates and structural similarities. These features facilitate cross-linguistic activation and account for the reliable priming effects observed in the Portuguese–English direction. The findings thus underscore typological distance as a decisive factor modulating semantic representation in trilinguals.

Beyond distance, the mediating role of L2 is equally critical in trilingual processing. Research has long demonstrated that L3 output is not only influenced by L1 but often more strongly shaped by L2 (De Angelis et al., 2009). Green’s (1998) inhibitory control model predicts that because L1 has the highest baseline activation, it requires stronger suppression to minimize interference with L2 and L3. When L3 activation is relatively weak, L2 emerges as the more salient competitor. Williams and Hammarberg (1998) termed this the “foreign language effect,” arguing that L2, as a non-native language, is cognitively closer to L3 and thus more likely to intrude during L3 production. Bardel and Falk’s (2007) “L2 status factor” hypothesis further refined this account, proposing that L2 and L3 share learning strategies and knowledge structures, granting L2 a privileged role in trilingual acquisition.

A growing body of evidence substantiates this framework. Bardel and Lindqvist (2007) showed that in Swedish learners of Italian (L3), L2 influence persisted consistently, whereas L1 played only a minor role. Bono and Melo-Pfeifer (2011) reported that over 60% of unintentional lexical intrusions in French speakers of Spanish (L3) originated from English L2. More recently, Stoehr et al. (2024), using a rapid trilingual switching paradigm with Spanish–Basque–English participants, found that L3 interfered more strongly with L2 than with L1, highlighting the robustness of L2–L3 connectivity.

The present findings provide direct evidence for this L2 mediation effect. The robust English → Chinese priming indicates that English (L2) functioned as a mediating pathway for activating Chinese (L3), while the absence of Portuguese → Chinese priming reflects the combined constraints of typological distance and acquisition sequence on L1–L3 interactions. In other words, although Portuguese is the native language, its substantial typological distance from Chinese limited direct semantic activation. By contrast, English, with higher proficiency, its role as the instructional medium for learning Chinese, and greater similarity to L3 in representational and learning strategies, conforms to the predictions of the L2 status factor and exerts a stronger mediating role in the trilingual semantic system.

These findings both support and challenge the Typological Proximity Model. On one hand, the absence of Portuguese ↔ Chinese priming validates TPM’s prediction that greater typological distance reduces cross-linguistic facilitation. On the other hand, the strong English → Chinese effect highlights an underexplored dimension: the privileged mediating role of L2 in linking typologically distant languages. Future models of trilingual processing must therefore integrate typological distance, psychotypology, and L2 status as dynamic factors shaping multilingual semantic representation.

In sum, typological distance and L2 mediation jointly shape the architecture of trilingual lexical representation. While distance constrains the probability and strength of cross-linguistic activation, L2 assumes a distinctive role in bridging distant systems. These findings deepen our understanding of trilingual lexical processing mechanisms.

## 6 Conclusion

The present study revealed a distinct asymmetrical pattern of cross-linguistic semantic activation among Portuguese–English–Chinese trilinguals. Significant priming was observed only in the Portuguese → English and English → Chinese directions, while no reliable effects emerged for Portuguese–Chinese or Chinese → Portuguese pairings. These findings demonstrate that L1 dominance cannot compensate for substantial typological distance, whereas L2, when functioning as both a higher-proficiency language and the instructional medium, can effectively mediate activation toward L3. This provides direct evidence that typological distance and language status jointly shape trilingual semantic representation.

Theoretical implications extend to models of multilingual processing. The absence of Portuguese–Chinese priming highlights typological distance as a categorical barrier that blocks automatic activation between structurally divergent systems, particularly when distinct writing systems are involved. Conversely, the reliable English → Chinese priming underscores the mediating role of L2, consistent with the L2 Status Factor hypothesis, which posits that L2 and L3 share closer cognitive and representational properties than L1 and L3. These findings challenge direct extensions of bilingual models such as the Revised Hierarchical Model and BIA+, underscoring the need for frameworks that integrate both typological compatibility and language status effects in trilingual contexts.

Beyond theoretical contributions, this study advances multilingual research by incorporating Chinese, a typologically underrepresented language. The logographic script, tonal phonology, and isolating morphology of Chinese establish processing pathways that diverge fundamentally from alphabetic Indo-European systems. The use of masked translation priming in a cross-family design represents a methodological innovation, demonstrating how activation thresholds are modulated when languages differ not only in lexicon but also in orthographic and morphological principles. These insights carry practical implications for multilingual education: leveraging L2 as a pedagogical bridge may facilitate L3 acquisition when L1 and L3 are typologically distant, as evidenced by English’s mediating role between Portuguese and Chinese.

Despite its contributions, this study has limitations. The findings are based on a specific trilingual group, and replication across different L1–L2–L3 configurations is necessary. Future research should examine whether similar patterns emerge in other cross-family contexts, explore neural correlates of typological distance, and track longitudinal changes as L3 proficiency develops.

## Data Availability

The original contributions presented in the study are included in the article/Supplementary material, further inquiries can be directed to the corresponding author/s.
