# Supplementary material for: Asymmetrical cross–linguistic semantic activation in Portuguese–English–Chinese trilinguals: evidence from masked translation priming
Source: Front Psychol. 2026 Jan 20;16:1734210. doi: 10.3389/fpsyg.2025.1734210 (PMC12864484; doi:10.3389/fpsyg.2025.1734210)
Supplement: Supplementary file 1 [file Data_Sheet_1.pdf]

Appendix 1: Masked Translation Priming Experiment Materials

Condition 1: Portuguese–English

L1 Portuguese (Prime)	L2 English (Target)	
**Related group (6 biological words; 6 non-biological words)**		
Amigo	Friend	
Motorista	Driver	
Escritor	Writer	
Gerente	Manager	
Cliente	Customer	
Laranja	Orange	
Filme	Movies	
Prato	Dish	
Pincel	Brush	
Mar	Sea	
Sumo	Juice	
Mochila	Backpack	
**Unrelated group (6 biological words; 6 non-biological words)**		
Menino	Grocer	
Marido	Shark	
Esposa	Doctor	
Ameixa	Seahorse	
Gato	Wolf	
Jasmim	Farmer	
Garrafa	Basket	
Escova	Frame	

L1 Portuguese (Prime)	L2 English (Target)	
Projetor	Bottle	
Quadro	Camera	
Câmera	Bottle	
Mesa	Shirt	

Condition 2: English–Portuguese

L2 English (Prime)	L1 Portuguese (Target)	
**Related group (6 biological words; 6 non-biological words)**		
Leaf	Folha	
Strawberry	Morango	
Pumpkin	Abóbora	
Eye	Olho	
Lamb	Cordeiro	
Monkey	Macaco	
Moon	Lua	
Stone	Pedra	
Sky	Céu	
Clothes	Roupa	
Kitchen	Cozinha	
Mirror	Espelho	
**Unrelated group (6 biological words; 6 non-biological words)**		
Bamboo	Pessoa	
Sheep	Homem	
Peach	Colega	
Whale	Escritor	

L2 English (Prime)	L1 Portuguese (Target)	
Lobster	Medico	
Violinist	Advogado	
Watch	Livro	
Shoe	Relogio	
Clothes	Carro	
Hairdryer	Telefone	
Spoon	Cadeira	
Clock	Porta	

Condition 3: Portuguese–Chinese

L1 Portuguese (Prime)	L3 Chinese (Target)	
**Related group (6 biological words; 6 non-biological words)**		
Joven	青年	
Ladrão	小偷	
Viajantes	旅客	
Advogado	律师	
Velho	老人	
Campeão	冠军	
Almoço	午餐	
Massa	面条	
Janela	窗户	
Avião	飞机	
Comboio	火车	
Lápis	铅笔	
**Unrelated group (6 biological words; 6 non-biological words)**		

L1 Portuguese (Prime)	L3 Chinese (Target)	
Pepino	小鸟	
Cavalo	大树	
Pássaro	司机	
Tubarão	工人	
Tulipa	男人	
Corvo	妻子	
Banco	菜单	
Igreja	地铁	
Colher	空调	
Selo	礼物	
Mercado	面包	
Teatro	面条	

Condition 4: Chinese–Portuguese

L3 Chinese (Prime)	L1 Portuguese (Target)	
**Related group (6 biological words; 6 non-biological words)**		
小鸡	Frango	
兔子	Coelho	
医生	Médico	
阿姨	Tia	
新娘	Noiva	
丈夫	Marido	
花园	Jardim	
盘子	Prato	
楼层	Andar	

L3 Chinese (Prime)	L1 Portuguese (Target)	
商店	Loja	
裤子	Calças	
桌子	Mesa	
**Unrelated group (6 biological words; 6 non-biological words)**		
坏人	Motorista	
鲜花	Gerente	
白猫	Cidadão	
熊猫	Amigo	
书本	Bombeiro	
护士	Sobrinho	
地图	Chave	
超市	Mochila	
蛋糕	Espelho	
果汁	Prato	
公园	Cama	
飞机	Vidro	

Condition 5: English–Chinese

L2 English (Prime)	L3 Chinese (Target)	
**Related group (6 biological words; 6 non-biological words)**		
Cabbage	白菜	
Eyes	眼睛	
Singer	歌手	
Bird	小鸟	
Child	孩子	

L2 English (Prime)	L3 Chinese (Target)	
Tree	树木	
Mirror	镜子	
Egg	鸡蛋	
Bread	面包	
Milk	牛奶	
Newspaper	报纸	
Book	书本	
**Unrelated group (6 biological words; 6 non-biological words)**		
Chicken	玉米	
Bear	观众	
Potato	同学	
Senator	葡萄	
Rose	经理	
Crocodile	大腿	
Company	啤酒	
Institution	太阳	
Machine	月亮	
Website	作业	
Blog	饮料	
Letter	篮球	

Condition 6: Chinese–English

L3 Chinese (Prime)	L2 English (Target)	
**Related group (6 biological words; 6 non-biological words)**		
西瓜	Watermelon	

L3 Chinese (Prime)	L2 English (Target)	
火鸡	Turkey	
男孩	Boy	
女人	Woman	
男友	Boyfriend	
叔叔	Uncle	
铅笔	Pencil	
天气	Weather	
菜单	Menu	
茶水	Tea	
米饭	Rice	
蛋糕	Cake	
**Unrelated group (6 biological words; 6 non-biological words)**		
水果	Spider	
医生	Dolphin	
孩子	Apple	
妹妹	Poet	
阿姨	Lawyer	
客人	Shrimp	
电话	Radio	
米饭	Bottle	
商店	Lamp	
医院	Computer	
房间	Stove	
宾馆	Napkin	
